# Evaluating Low‐Frequency Ultrasound as a Pretreatment to Improve Ozonation Antimicrobial Efficacy in Urban Wastewater Treatment

**DOI:** 10.1002/wer.70322

**Published:** 2026-02-25

**Authors:** Alessandro Moretti, Elisabetta Gover, Giulia Bisson, Clara Comuzzi, Daniele Goi, Marilena Marino

**Affiliations:** ^1^ Polytechnic Department of Engineering and Architecture University of Udine Udine Italy; ^2^ Department of Agricultural, Food, Environmental and Animal Sciences University of Udine Udine Italy

**Keywords:** inactivation kinetics, low‐frequency ultrasound pretreatments, ozone, wastewater antimicrobial treatment

## Abstract

Technologies based on oxidation processes, particularly ozonation, have shown great potential for microbiological wastewater (WW) treatment. Applying low‐frequency ultrasound (LF‐US) can cause sublethal damage to microbial cells by generating nanobubbles, potentially enhancing their sensitivity to ozonation. Thus, primary urban WWs spiked with *Salmonella enterica* , *Escherichia coli* , *Enterococcus faecium* , and *Pseudomonas fluorescens* were subjected to laboratory‐scale ozonation with or without LF‐US pretreatment. LF‐US pretreatment increased the antimicrobial efficiency of ozonation for all targets, with the most significant increase of 5.16 ± 0.17 Log CFU/mL for *P. fluorescens* (LF‐US 600 s followed by ozone 120 s) and 0.57 ± 0.08 Log CFU/mL for *Ent. faecium* (LF‐US 300 s followed by ozone 30 s). By comparing individual processes with the combined treatment and using inactivation curves from laboratory experiments, it was estimated that ozone treatment following short LF‐US pretreatments saved 67 ± 9% of energy and reduced costs by up to 15.1 ± 1.3 €/m^3^ of treated water. This highlights the potential of this sequential method for effective and cost‐efficient WW sanitation.

## Summary

- Ozonation significantly reduces the microbial viability in wastewater.
- Pretreatments with low‐frequency ultrasound improve the effect of ozonation.
- Weibull‐tail inactivation modeling supports the identification of energy‐ and cost‐efficient treatment conditions.

## Graphical abstract

In urban wastewater contaminated with *Salmonella enterica* , *Escherichia coli* , *Enterococcus faecium* , and *Pseudomonas fluorescens* , a low‐frequency ultrasound pretreatment enhanced the antimicrobial effectiveness of ozonation. Additionally, pretreatment led to energy and cost savings compared to ozonation alone, highlighting the potential of this sequential method for efficient and economical wastewater sanitation.

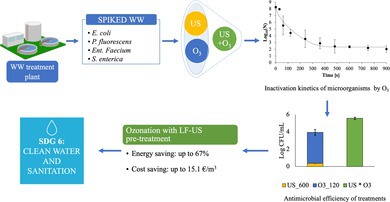

## 1 Introduction

The continuous rise in population, climate change, and the overuse of high‐quality water may result in a severe global water pollution crisis. Over the past century, water usage has grown twice the population growth rate (McBean 2016). Projections indicate a 55% increase in water demand by 2050, driven by manufacturing, electricity generation, and household needs (Boretti and Rosa 2019). Countries worldwide must prepare to meet the United Nations Sustainable Development Goal 6, which targets comprehensive and sustainable water management and sanitation by 2030, in light of predicted water exploitation trends (Dilekli and Cazcarro 2019). Achieving this goal presents a significant opportunity through treating and recovering wastewater (WW), necessitating substantial research and technological investment.

Disinfection processes are crucial in the context of WW recovery. Pathogenic microorganisms, such as bacteria, viruses, and protozoa, can spread through water, and ingesting contaminated water may lead to increased gastrointestinal diseases. As a result, ensuring effective disinfection is essential for maintaining suitable public health standards (Levantesi et al. 2010). In 2023, 42 outbreaks, involving 1954 people, were reported in Europe due to the consumption of water contaminated with biological agents, including *Campylobacter*, *Clostridium perfringens* , Hepatitis A virus, *Escherichia coli* , norovirus, and *Salmonella* (EFSA/ECDC 2023). Around the world, it is estimated that 1.8 million people annually are at high risk of death due to infections that have water as a vehicle (Sorensen et al. 2021). In addition, the water flows frequently contain bacteria resistant to antibiotics, one of the most pressing health emergencies worldwide. The six leading antibiotic‐resistant pathogenic bacteria have been estimated to have caused over 900,000 deaths in 2019 (Murray et al. 2022).

Chemical disinfection, especially chlorination, is one of the most adopted methods for reducing the presence of microorganisms in water, thanks to its ease of use and reduced costs. However, chlorine reacting with organic matter can generate hazardous disinfection by‐products. Moreover, chlorination can lead to the regrowth or reactivation of bacteria, including antibiotic‐resistant species (Huang et al. 2011; Kali et al. 2021). Alternative effective disinfection methods for WW include using biocides, but they can lead to the selection of resistant microbial species. Heat treatments and UV light are also effective, but they can cause the alteration of the organoleptic properties and high costs, making them unattractive (Lena et al. 2024; Meireles et al. 2016).

Nowadays, unconventional practices in liquid waste and WW treatment are the processes able to generate reactive oxygen species (ROS). ROS are strong oxidant radicals that lethally affect many microorganisms (Comuzzi et al. 2020; Hussain et al. 2022). Among such processes, ozonation proved very effective against microorganisms. Ozone is a potent oxidant widely studied and applied in various WW treatment plants. Because of its ability to produce free radicals, ozone is a powerful disinfectant. However, ozone has low solubility in water, which may necessitate expensive plant solutions to increase efficiency, such as adjusting the pH or controlling the ozone flow and the size of the diffusion bubbles inside the ozone disinfection reactors. Additionally, certain conditions can lead to the formation of toxic by‐products (e.g., aldehydes), requiring the identification of new treatment solutions (Wang and Chen 2020).

Other treatments, coupled with ozone, could help mitigate the by‐product formation while maintaining high antimicrobial efficacy. For instance, the combined use of photocatalysis and ozonation resulted in 50%–75% faster inactivation of *E. coli* , *Salmonella*, *Shigella*, and *Vibrio cholerae* in intentionally contaminated water compared to the use of ozonation alone (Mecha et al. 2017). Ozone with UV light and chlorine also synergistically affected microbial inactivation (Shi et al. 2021). Again, combining high‐frequency ultrasound (US) and ozone might result in higher bacterial inactivation rates. Because of US simultaneous treatment, the transfer rate of ozone into the liquid matrix is increased because of the generation of radicals (Guo et al. 2015; Yargeau and Danylo 2015). Such treatments effectively reduced the viability of coliforms, *E. coli* , and enterococci in WW (Al‐Hashimi et al. 2015; Chen et al. 2021; Rossi et al. 2021). However, there is a lack of data for other bacteria, such as *Pseudomonas* and *Salmonella*, that are often found in WW, indicating fecal contamination or potential regrowth, as well as raising concerns about antibiotic resistance (Levantesi et al. 2012; Nguyen et al. 2021; Ribas et al. 2000).

When US is applied at low frequencies (LF‐US), the cavitation phenomenon can cause sublethal damage to the membrane of microbial cells, making them more susceptible to subsequent treatment (Li et al. 2017). Unlike high‐frequency US, which often produces heat and strong radical formation leading to direct microbial inactivation, LF‐US mainly generates mechanical cavitation. This results in reversible membrane perturbations, nanobubble formation, and enhanced mass transfer without substantially reducing microbial viability on its own (Sundaram et al. 2003). Such a mild, nondestructive action makes LF‐US particularly suited as a conditioning step that can increase the effectiveness of oxidizing treatments by facilitating the penetration of reactive species. This strategy has potential applications in food (Nicolau‐Lapeña et al. 2019) but remains unexplored in an environmental context. Thus, this study aimed to evaluate the effect of LF‐US pretreatments on the microbial sensitivity to ozone in WW. To this aim, spiked WW with *E. coli* , *Pseudomonas fluorescens* , *Enterococcus faecium* , and *Salmonella enterica* were ozonated with or without an LF‐US pretreatment, and the kinetics of microbial survival were evaluated. Furthermore, an assessment of cost and energy effectiveness was carried out from a scaling‐up perspective.

## 2 Materials and Methods

### 2.1 WW

The WW was collected from the primary effluent of the urban WW treatment plant in Udine (100,000 Population Equivalent), in the North‐East of Italy. The WW characteristics were similar to typical urban treatment plant inflows (Van de Walle et al. 2023). Before each trial, WW was sterilized at 121°C for 15 min to remove contaminants, ensuring no interference and standardizing the matrix. Before and after sterilization, chemical oxygen demand (mg/L), specific ultraviolet absorbance (L/mg m), total nitrogen (mg/L), total phosphates (mg/L), anionic surfactants (mg/L), and pH were assessed (APHA 2023).

### 2.2 Microbial Strains

WW samples were spiked with *P. fluorescens* (strains CECT 378, DIAL22, and DIAL049), *E. coli* (strains DSM 1116, DIAL4315, and DIAL1411), *S. enterica* subsp. *arizonae* (strains DSM 9386, DIAL40, and DIAL52), and *E. faecium* (strains DSM 20477, DIAL2146, and DIAL040). The strains were identified by partial 16S rRNA gene amplification (Innocente et al. 2019) and stored at −80°C in Brain Heart Infusion (BHI) broth (Oxoid, Milan, Italy). For each species, trials were performed with a pool of three strains.

### 2.3 Sample Preparation

The strains were grown overnight in BHI incubated at 30°C (for *P. fluorescens* ) or 37°C (for *E. coli* , *Ent. faecium*, and *S. enterica* ). Then, the cultures of the same microbial genus were pooled, and the cells were recovered by centrifugation at 4000 × *g* for 10 min. Cells were washed twice and resuspended in 4 mL of Maximum Recovery Diluent (MRD) (Oxoid). The suspension was used to inoculate 4 L of WW for each microbial pool (final viable count about 10^7^–10^8^ CFU/mL).

### 2.4 LF‐US Treatments

A 250‐W processor (Hielscher Ultrasonics, Teltow, Germany) with an ultrasonic transducer (working frequency equal to 24 kHz) and a 22‐mm Ø BS24 sonotrode was used. In each trial, 200 mL of spiked WW was treated for 5 s (name of the treatment US_5 from here on), 10 s (US_10), 30 s (US_30), 60 s (US_60), 90 s (US_90), 180 s (US_180), 300 s (US_300), 450 s (US_450), and 600 s (US_600). The treatments were conducted at 18°C and at 20% of the maximum amplitude to optimize the ratio between sample disinfection and absorbed power, with an average absorbed power of 74 W. The latter was continuously measured throughout the entire treatment process thanks to a power meter connected to the US generator plug. The US duty cycle was 100% (continuous treatment) with a probe depth of 5 cm.

### 2.5 Ozonation Treatments

An open CL‐010‐DT ozone generator (AirTree Ozone Technology Co., Sijhih, Taiwan) was used. The real absorbed power was measured through a power meter connected to the ozone generator plug, revealing a 135‐W average value. Ozone was bubbled directly into 200‐mL WW samples at 18°C in a batch mode (2 L/min of airflow, atmospheric air feed) through a tube connected to a bottom diffuser (Figure S1). The generator, with external air as the gas feed, pumped the produced ozone into the reactor containing WW via a bottom diffuser. To work in controlled environments with hazardous gases, the excess ozone that was not dissolved or utilized during treatment was captured by a KI trap, allowing the residual ozone to flow into the second reactor. Anyway, all the experiments were performed under a fume hood. The ozone production at 2‐L/min airflow was set at 1.2 g/m^3^, revealing an overall ozone generator efficiency of 1.07 g/kWh. The calculated ozone transfer efficiency (TE) in water was around 72%, fed by an initial gas flow rate of 2 g/h. High TE values (> 90%) are reportedly associated with pressurized reactors equipped with controlled fine‐bubble diffusers (Rakness et al. 2018). Ozonation was carried out for 30 s (name of the treatment O3_30 from here on), 60 s (O3_60), 120 s (O3_120), 240 s (O3_240), 360 s (O3_360), 480 s (O3_480), 600 s (O3_600), 750 s (O3_750), and 900 s (O3_900).

### 2.6 Ozonation With LF‐US Pretreatments

Spiked WWs (200‐mL aliquots) were treated with a LF‐US pretreatment followed by ozone, connected in series. Thirty‐, 90‐, 300‐, and 600‐s US treatments were applied on 200‐mL WW samples along with two ozone treatments for each microbial target (60 and 120 s for *P. fluorescens* , 30 and 60 s for *Ent. faecium*, 15 and 30 s for *E. coli* , and 30 and 45 s for *S. enterica* ). The treatment labeling involved pairing the name of the US treatment with the name of the ozone treatment, separated by an asterisk. For example, the treatment sequence of US for 30 s followed by ozone for 60 s was denoted as US_30*O3_60. In any case, the experimental setup and the calibration of both single and combined processes were designed to optimize treatment efficiency in terms of microbial inactivation and energy demand. Several combinations of US amplitude, ozone airflow, and operating temperature were evaluated, and the conditions adopted in this study were identified as the most effective. For example, increasing the US amplitude resulted in substantially higher energy consumption without a proportional improvement in microbial inactivation, and it also led to unavoidable temperature rises that could negatively influence subsequent ozone dissolution in water. Similarly, higher ozone airflow rates produced greater ozone volumes, but excessive bubble formation impaired mass transfer, generating larger bubbles that reduced gas solubility and ultimately decreased the concentration of dissolved ozone in the system. Lastly, the selected operating temperature avoided the need for additional cooling steps—an energy‐intensive requirement—while maintaining suitable conditions for ozone treatment, as ozone solubility in water is strongly dependent on temperature.

### 2.7 Microbiological Analysis

WW samples were decimally diluted in MRD and analyzed using the drop plate technique on BHI plates (Herigstad et al. 2001) incubated for 24 h at 37°C (*Ent. faecium*, *E. coli* , and *S. enterica* ) or 30°C ( *P. fluorescens* ). The limit of detection (LOD) for this method was 20 CFU/mL.

### 2.8 Modeling of Inactivation Kinetic

All experiments were conducted in at least two biological replicates, and the results were presented as mean ± standard deviation (SD). The means were subjected to ANOVA (*p* < 0.05).

For samples exposed only to ozone, viable counts were plotted against exposure time and modeled using the Excel add‐in GinaFiT using the Weibull with the tail distribution (Albert and Mafart 2005; Geeraerd et al. 2005) shown in Equation (1):

(1) $$N=\log_{10}\left[\left(10^{\log_{10}\left(N_{o}\right)-}10^{\log_{10}\left(N_{\mathrm{res}}\right)}\right)\times 10^{\left(-{\left(\frac{t}{\delta }\right)}^{\varepsilon }\right)}+10^{\log_{10}\left(N_{\mathrm{res}}\right)}\right]$$

where *N* is the microbial concentration (CFU/mL), *N*_0_ is the initial concentration (CFU/mL), *N*_*res*_ is the residual microbial concentration (CFU/mL), *t* is the time (s), *ε* is the shape parameter (> 1 for concave curves or < 1 for convex curves), *δ* is the time required to obtain the first decimal reduction (s).

GInaFiT provides point estimates for *δ*, *ε*, and *N*_*res*_ together with goodness‐of‐fit indicators (*R* ^2^). Additionally, the model estimates the 4‐D value (s), which is the time required to achieve four logarithmic reductions of *N*_0_.

### 2.9 Assessment of Energy and Cost‐Effectiveness

The *E*_US*O3_ (kWh) was calculated using Equation (2) for the US and ozone energy absorption per cubic meter:

(2) $$E_{\mathrm{US}*O_{3}}=E_{\mathrm{US}}+E_{O_{3}}=\frac{P_{\mathrm{US}}\;t_{\mathrm{US}}}{V}+\frac{\;P_{O_{3}}\;t_{O_{3}}}{V}$$

where *P*_US_ and *t*_US_ are the absorbed power of the US generator (kW) for a given time (h), *P*_O3_ and *t*_O3_ are the absorbed power of the ozone generator during a given time *t*_O3_ application, and *V* is the maximum volume (m^3^) treated in 1 h.

The energy use per cubic meter ${E_{O_{3}}}^{*}$ was calculated according to Equations (3) and (4):

(3) $${E_{O_{3}}}^{*}=E_{O_{3}}+∆E_{O_{3}}$$

(4) $$∆E_{O_{3}}=\frac{\;P_{O_{3}}∆t_{O_{3}}}{V}$$

where $∆t_{O_{3}}$ is the additional time needed for ozonation to gain a logarithmic reduction equal to that obtained after the ozonation treatment with LF‐US pretreatment.

To assess the cost‐effectiveness of the ozonation with LF‐US pretreatment, the energy saving ($E_{s\%}$) was calculated (Equation 5):

(5) $$E_{s\%}=1-\frac{E_{\mathrm{US}*O_{3}}}{\;E_{\mathrm{US}}+{E_{O_{3}}}^{*}}$$

The costs of the ozonation with LF‐US pretreatment ($M_{\mathrm{US}*O_{3}}$), US ($M_{\mathrm{US}}$), and ozonation ($M_{{O_{3}}^{*}}$) treatments were expressed in €/m^3^ and calculated, respectively, through Equations (6), (7), and (8):

(6) $$M_{\mathrm{US}*O_{3}}=\;E_{\mathrm{US}*O_{3}}*C_{\text{€}}$$

(7) $$M_{\mathrm{US}}=\;E_{\mathrm{US}}*C_{\text{€}}$$

(8) $$M_{{O_{3}}^{*}}=\;{E_{O_{3}}}^{*}*C_{\text{€}}$$

The total savings were then calculated (Equation 9):

(9) $$∆M=\left(M_{\mathrm{US}}+M_{{O_{3}}^{*}}\right)-M_{\mathrm{US}*O_{3}}$$

where $C_{\text{€}}$, which is the standard purchasing price for a kWh within the considered Italian case study, equals 23.4 c€/kWh (ARERA 2021). It was assumed that the processes ran continuously to estimate the annual energy consumption cost.

## 3 Results and Discussion

The study reports assessing the potential ozonation, applied alone or after an LF‐US pretreatment, to recover WW from a microbiological perspective. Additionally, the study includes energy balances and cost analyses.

### 3.1 LF‐US Treatments

In this study, sterilized WW was artificially contaminated with specific microbial targets, and the effects of LF‐US treatments were evaluated. Sterilization of WW can modify its physicochemical characteristics; therefore, several parameters were assessed in this regard in WW before and after sterilization (Table S1). Despite the reduction in concentration caused by sterilization, most parameters remained within the range typically observed in urban WW. In contrast, specific ultraviolet absorbance increased after sterilization; however, the final values remained consistent with an organic matter content unlikely to promote the formation of disinfection by‐products (Sharma et al. 2021).

**FIGURE 1:**
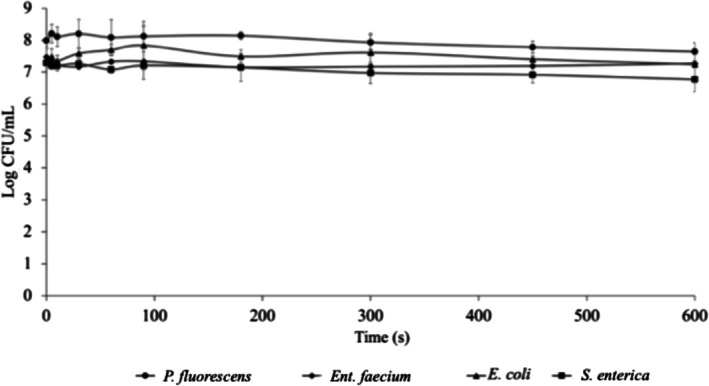
Mean viable counts (Log CFU/mL ± SD) after LF‐US treatments. Data represent mean ± SD of *n* = 2 biological replicates. Error bars indicate standard deviation.

Spiked WWs were subjected to single LF‐US treatments, and the effect on microbial targets was assessed. The samples were treated at 24 kHz, and the residual viability as a function of exposure time was evaluated for each bacterial genus. High‐frequency US is known to generate extreme temperature and pressure gradients, which can lead to microbial inactivation through thermal effects, high‐velocity microjets, and free‐radical formation (Gibson et al. 2009). In contrast, low‐frequency US produces larger cavitation bubbles and more intense mechanical collapses, making it attractive for enhancing mass transfer or weakening cell structures in combined processes. However, LF‐US does not necessarily provide effective disinfection on its own, particularly when operated under mild, temperature‐controlled conditions. For this reason, in this study, LF‐US was used at a frequency of 24 kHz and a maximum power of 74 W for 5–600 s, keeping the temperature constant at about 18°C to limit the antimicrobial effect to cavitation only and not to temperature rise, which can occur during US treatments (Chemat et al. 2017). As expected, only a minor, nonstatistically significant decrease in microbial viability was observed, even after the longest treatment (Figure 1). When exposed to stronger treatments (100 W for 60 min), *E. coli* showed a Log reduction of less than 1 (Al‐Hashimi et al. 2015). Similar data were also reported for other Gram‐positive and Gram‐negative bacteria (Chen et al. 2021). Instead, a reduction of up to 7 Log CFU/mL was achieved by using a high‐frequency US (850 kHz) or by applying the treatment without temperature control, which allows a temperature of about 90°C to be reached (Gao et al. 2014; Vázquez‐López et al. 2019).

### 3.2 Ozone Treatments

**FIGURE 2:**
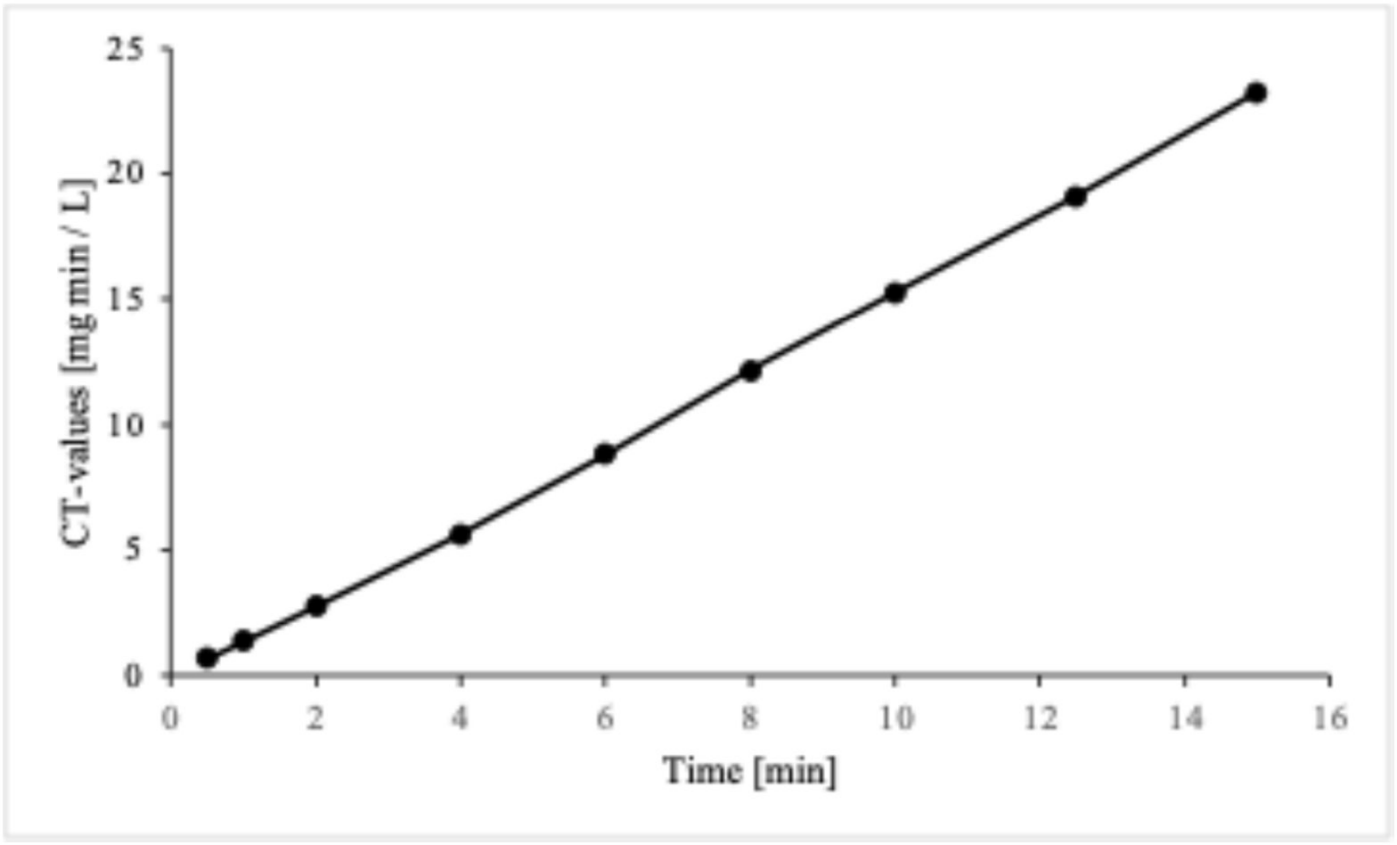
Ozone CT‐values.

Spiked WW samples were treated with ozone for up to 900 s. The mean dissolved ozone concentration in liquid resulted to be equal to 1.46 mg/L throughout the entire duration of the experiments. The dissolved concentration of ozone was measured through the indigo method (Gordon et al. 2000). For a better understanding of the ozonation process, contact time (CT) values are reported in Figure 2, for the entire exposure time tested (0.5–15 min).

**FIGURE 3:**
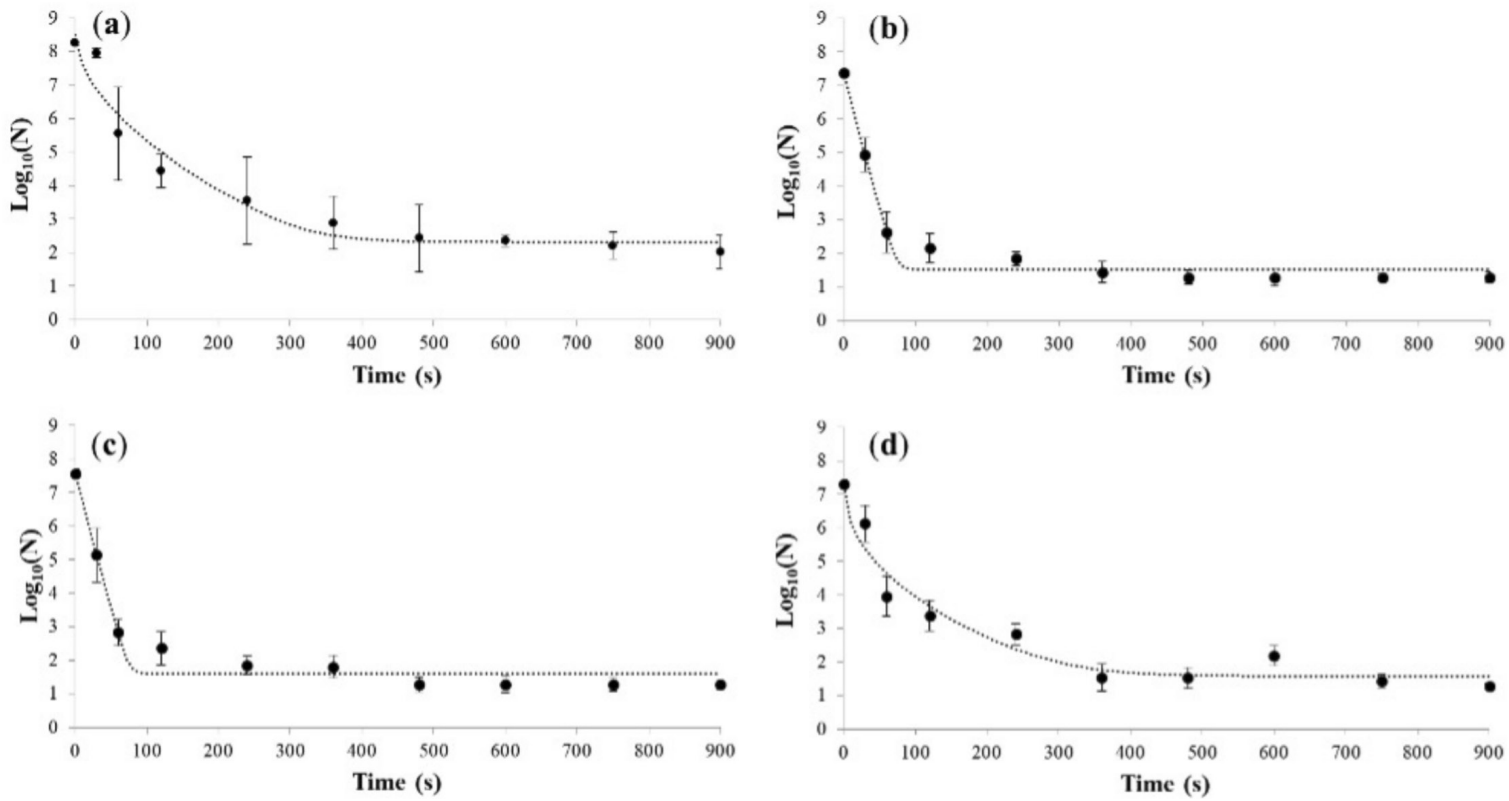
(a) *P. fluorescens* , (b) *Ent. faecium*, (c) *E. coli* , and (d) *S. enterica* inactivation (Log CFU/mL ± SD) in spiked WW by ozonation treatments; the dotted line represents data fitted according to the Albert and Mafart model. Data represent mean ± SD of *n* = 2 biological replicates. Error bars indicate standard deviation.

In this study, the viable count decreased for all microbial targets as the treatment time increased. The inactivation kinetics were then analyzed using the GInaFiT software. Based on the *R* ^2^ values obtained, the best‐fit inactivation model was the Albert and Mafart model (Figure 3). This model characterizes the inactivation kinetics with a convex, concave, or linear curve followed by a tailing effect (Albert and Mafart 2005). Ozone is a powerful disinfectant with strong bactericidal activity. The mechanism of O_3_ involves two processes: the direct oxidation of compounds by the O_3_ molecule and an indirect reaction that includes the radical products formed during the decomposition of O_3_, specifically hydroxyl radicals (Saravanan et al. 2021). It is widely used in medical, agricultural, marine, and food industries to inactivate suspended and surface‐attached microorganisms (Guo and Wang 2017; Lazarova et al. 2013; Marino et al. 2015). Although ozone can also find a practical application for the antimicrobial treatment of WW, studies on inactivation kinetics are only a few (Czekalski et al. 2016; Lee et al. 2016). It should be noted that modeling the inactivation kinetics of microorganisms is relevant to describing and predicting the efficacy of any antimicrobial treatment.

The inactivation kinetics showed a convex pattern, as suggested by the estimated *ε* values, which were all < 1 (Table 1). The data showed that when treating WW with ozone, there was a pattern of inactivation with a convex shape followed by a tail. This observation suggested that all target cells had the same resistance at the beginning of the treatment. Still, there may be a subpopulation of more resistant cells, indicated by the presence of the tail. Inactivation of the microbial targets occurred mainly in the first few minutes of treatment. The values estimated by the model showed the time required to kill 90% of the bacterial cells (*δ*), expressed as the decimal reduction time of the microbial load. The most sensitive organism was *S. enterica* , with 90% of cells killed after just 6 s of ozonation. As for *Ent. faecium*, *E. coli* , and *P. fluorescens* , *δ* values ranged from 12.04 to 15.71 s, which underlines the excellent efficiency of ozonation as an antimicrobial strategy for WW. *P. fluorescens* was the most challenging microbial target to inactivate through ozonation. A 4‐D inactivation was achieved after 162 s. Bacteria belonging to the genus *Pseudomonas* are known to produce several antioxidant enzymes, including superoxide dismutase, catalase, and peroxidase, which may provide an essential line of defense against ROS (Fones and Preston 2012). Also, exopolysaccharides, which cover the outer surface of the cell of this microbial genus, and the intracellular accumulation of polyesters, such as polyhydroxyalkanoates, can play a pivotal role in counteracting oxidative damage (Ayub et al. 2009; Chang et al. 2007). Future studies will include targeted analyses to experimentally confirm the role of these mechanisms in *Pseudomonas* oxidative‐stress resistance. Based on the data collected and the inactivation kinetics of ozonation, it was observed that a portion of the microbial population survived the treatments for all microbial targets. Considering the high rate of microbial multiplication and the potential risks associated with water contaminated by pathogenic microorganisms, further intervention is necessary to lower the health risk. For this reason, we tested the effect of pretreatment with US on subsequent inactivation with ozonation.

**TABLE 1:** Model parameters (mean ± SD) and regression coefficient estimated by fitting Albert and Mafart's model on the reduction of spiked WW counts as a function of time for ozonation treatments. *ε* = shape parameter, *δ* = time required to obtain the first decimal reduction, 4‐D = time required to obtain 4‐Log reductions of the initial population.

	*ε*	*δ* (s)	4‐D (s)	*R* ^2^
*P. fluorescens*	0.56 ± 0.17	15.71 ± 1.41	162	0.96
*Ent. faecium*	0.68 ± 0.20	14.68 ± 2.94	54	0.97
*E. coli*	0.98 ± 0.31	12.04 ± 1.37	54	0.97
*S. enterica*	0.44 ± 0.13	6.18 ± 1.16	144	0.95

### 3.3 Ozonation With LF‐US Pretreatments

**FIGURE 4:**
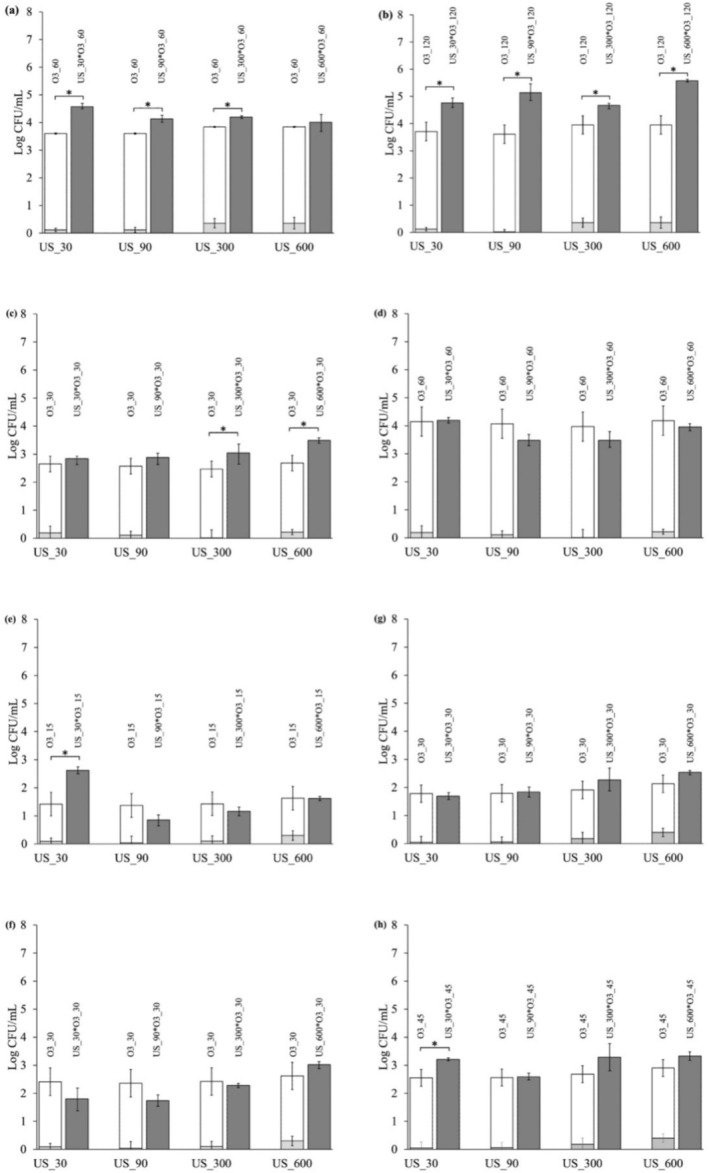
Log reduction (mean ± SD) of (a, b) *P. fluorescens* , (c, d) *Ent. faecium*, (e, f) *E. coli* , and (g, h) *S. enterica* viability following LF‐US (light gray bar), ozone (white bar), and ozonation with LF‐US pretreatments (dark gray bar). Asterisks mean a statistical difference between the sum of LF‐US and ozone applied alone and sequentially. Data represent mean ± SD of *n* = 2 biological replicates. Error bars indicate standard deviation. Statistical analysis was performed using one‐way ANOVA (*p* < 0.05).

LF‐US and ozone were sequentially applied to spiked WW. Four LF‐US treatments were carried out, namely, US_30, US_90, US_300, and US_600 s. As for ozonation, two different time intervals were selected for each microbial genus based on the time required to achieve the first decimal and 4‐D reductions in the initial viable count. Therefore, these times vary based on each tested microbial strain, requiring the employment of different ozone durations. The selected treatments were O3_60 and O3_120 for *P. fluorescens* , O3_30 and O3_60 for *Ent. faecium*, O3_15 and O3_30 for *E. coli* , and O3_30 and O3_45 for *S. enterica* , respectively (Figure 4). In this study, the treatments with LF‐US and ozone applied sequentially yielded endpoint reductions rather than full inactivation curves; therefore, no kinetic modeling was performed for the combined treatments. Future studies will generate complete survival curves for the sequential treatments, enabling mechanistic modeling and a more detailed interpretation of the sonozone interaction.

Many treatments significantly decreased the viability of *P. fluorescens* compared with the sum of LF‐US and ozonation alone, suggesting an enhanced combined effect. Except for treatment US_600*O3_60, the increased loss of viability in the treatments ranged from 0.36 to 1.63 Log CFU/mL. US_600*O3_120 was the most effective treatment, killing 5.58 Log CFU/mL of *P. fluorescens* . Overall, LF‐US pretreatments applied just before ozonation proved to be very effective against *P. fluorescens* despite the slight loss of viability following the LF‐US treatment alone, probably due to LF‐US enhancing the permeability of the outer membrane (Runyan et al. 2006). As for *E. coli* , viability loss was significantly improved when ozone was used after LF‐US in treatment US_30*O3_15 (*p* < 0.05). Although the LF‐US treatment proved relatively ineffective, even with prolonged application, it could still trigger morphological and functional alterations that render cells more vulnerable to subsequent treatments. US can disaggregate bacterial clusters into single cells, making them more ozone‐sensitive. In addition, the collapse of bubbles that form by cavitation breaks chemical bonds within the bacterial membrane, which weakens the cells against oxidative stresses (Al‐Hashimi et al. 2015). In a study carried out on naturally contaminated WW, authors ascribed the enhanced killing efficiency of a combined sonozone process to the US‐related release of the entrapped bacterial cells from the suspended particles, wherein they could be hidden, thus allowing ozone‐killing activity to be more effective (Rossi et al. 2021). In Gram‐negative bacteria, US initially targets the outer membrane, causing cellular injury, as was demonstrated for *E. coli* (Ananta et al. 2005). As treatment progresses, it becomes multitargeted, affecting the cell wall, cytoplasmic membrane, and internal structure (Li et al. 2016). It can be hypothesized that the LF‐US treatment induced sonoporation, leading to increased membrane permeability and facilitating ozone access to intracellular components (Dai et al. 2023). The findings of this study are significant, given that *Pseudomonas* and *E. coli* are major health‐related microbial contaminants in WW (Devatha and Pavithra 2019; Lee et al. 2016).

Regarding *Ent. faecium*, the only treatments that demonstrated an enhanced loss of viability compared with pure ozone treatments were those involving US for 300 and 600 s followed by ozonation for 30 s, increasing loss of viability of 0.57 and 0.81 Log CFU/mL, respectively (Figure 4). The ozone treatments for 60 s did not enhance the bactericidal effect compared with the sum of the individual treatments. This observation suggests that the microbial reaction to US may entail structural damage and gene expression changes. Studies have shown that when bacteria are exposed to US below their tolerance threshold, mechano‐transduction can occur, triggering the bacterial stress response (Murphy et al. 2016). LF‐US treatments can reduce gene expression in the citric acid cycle, respiratory chain, and ABC (ATP‐Binding Cassette) transporters, leading to decreased ATP production and reduced membrane permeability (Li et al. 2021). Nevertheless, following such treatment, bacteria initiate DNA and protein repair mechanisms, which could elucidate the observed relative tolerance of *Enterococcus* (Chapman et al. 2013).

The viability of *S. enterica* was notably decreased by the US_30 followed by O3_45 treatment compared with when US and ozone were applied individually. Micropores generated by LF‐US have not significantly affected *Salmonella* viability, allowing most cells to survive and grow in culture media after treatment (Kong et al. 2024). However, in our study, pretreatment was shown to enhance the effect of ozone, so some form of sublethal damage can be assumed. Although the ozone and US treatments have been documented to inactivate *Salmonella*, thus enhancing the safety of plant foods like cabbage, spinach, and lettuce (Siddique et al. 2021; Sun et al. 2022; Traore et al. 2020), there is currently no available literature on their application in WW treatment, to the best of our knowledge.

Although the strains used in this study represent microbial groups commonly found in WW, they do not encompass the full range of environmentally persistent or stress‐adapted bacteria. Future studies will therefore expand the strain panel to include isolates with enhanced environmental resilience, such as chlorine‐tolerant strains, biofilm‐forming variants, or taxa known for elevated oxidative‐stress resistance, to increase the practical relevance of treatments under real WW conditions.

### 3.4 Energy and Cost‐Effectiveness Analysis

The design and optimization of a process require an assessment of energy consumption and costs to achieve a reliable, economically feasible technology. The laboratory tests produced remarkable outcomes in microbial inactivation for specific treatments. For treatments where LF‐US was demonstrated to enhance ozone's antimicrobial activity, an assessment was conducted to determine the additional ozonation time required to achieve the same antimicrobial effect as ozonation with LF‐US pretreatment. This estimation was facilitated by studying the microbial inactivation kinetics. Considering the above, the ozone treatment duration necessarily differs among strains due to the peculiar resistance of each microbial target to the inactivation methods applied. Therefore, specific exposure times were selected for each strain to cover the interval between δ and 4‐D (Table 1). Then, to compare single ozonation with the combined technique, the hypothetical exposure time (derived from the inactivation curves) was evaluated, corresponding to the condition in which the sum of the reductions achieved by the individual treatments (US and ozone) equaled the reduction obtained by the combined US and O_3_ treatment. To achieve this, a ∆t_(O_3) (Equation 4) was introduced. Based on these data, estimates of the annual energy savings (*E*_s%_) and total cost savings (∆*M*) were made, assuming continuous application of the processes (Table 2).

**TABLE 2:** Energy‐ and cost‐effectiveness (mean ± SD) of specific sonozone treatments.

	Treatment	*E*_s%_	Δ*M* (€/m^3^)
*P. fluorescens*	US_30*O3_60	67 ± 9%	12.0 ± 1.0
	US_90*O3_60	55 ± 3%	9.0 ± 0.8
	US_300*O3_60	43 ± 5%	9.5 ± 0.8
	US_30*O3_120	42 ± 3%	8.1 ± 0.7
	US_90*O3_120	46 ± 4%	11.0 ± 0.9
	US_300*O3_120	30 ± 2%	7.5 ± 0.6
	US_600*O3_120	38 ± 2%	15.1 ± 1.3
*Ent. faecium*	US_300*O3_30	15 ± 4%	1.8 ± 0.2
	US_600*O3_30	11 ± 8%	2.1 ± 0.2
*E. coli*	US_30*O3_15	43 ± 16%	1.5 ± 0.1
*S. enterica*	US_30*O3_45	43 ± 5%	3.5 ± 0.3

Regarding energy savings, the application of US pretreatment improved energy efficiency from 30% to 67%, depending on the type of microorganism and the treatment time. The cost savings per cubic meter of treated WW varied between € 1.5 and € 15.1. However, this was not directly proportional to the *E*_s%_ value, as it depended on treatment time and the intrinsic resistance features of each microbial target. Cost and energy efficiency evaluations of combined treatments for removing chemical contaminants such as phenol, azo dye, and trichloroethylene have been documented in the literature (Mahamuni and Adewuyi 2010; Rekhate and Srivastava 2020). However, to our knowledge, no similar evaluations have been conducted for microbial contaminants. A comprehensive assessment of antimicrobial treatments' economic and energy efficiency on WW must also consider additional factors, such as the treatment and maintenance plant costs, which vary based on the maximum WW flow rate. Nevertheless, the kinetic approach used in this study can serve as a valuable starting point for evaluating the feasibility of unconventional WW treatments.

## 4 Conclusion

In this study, LF‐US applied before ozonation significantly enhanced the inactivation effect on specific microbial targets relevant to public health and antimicrobial resistance. After reducing the microbial load, treated WW can be reused in several ways, e.g., agricultural irrigation, industrial processes, and landscape irrigation, providing both environmental and economic benefits. The kinetic analysis of microbial inactivation allowed for assessing the energy and financial aspects of sonozone treatments, which is crucial for large‐scale WW disinfection. As this was an exploratory study primarily focused on microbial inactivation, the behavior of antibiotic‐resistance genes (ARGs) under LF‐US and ozonation was not investigated. Although ozone may contribute to nucleic‐acid damage and LF‐US can induce sublethal membrane alterations, the extent to which the combined treatment affects ARG persistence or potential regrowth remains to be established. Although beyond the scope of the present work, future studies will specifically address ARG quantification and posttreatment regrowth dynamics to better clarify these aspects. Similarly, the formation of ozonation by‐products was not assessed; future work will include their characterization to ensure that chemical side effects do not offset microbiological benefits. Although this work represents an initial investigation and was conducted with limited biological replicates, the findings highlight the potential of combining ozonation with LF‐US pretreatment. Further experiments under different conditions and scaling‐up are necessary to determine if such processes could be a viable alternative technology for future WW treatment. The simplified approach could be validated using ozonation with LF‐US pretreatment in a disinfection process line.

## Author Contributions

**Alessandro Moretti:** conceptualization, methodology, validation, investigation, formal analysis, writing – original draft, writing – review and editing, visualization. **Elisabetta Gover:** methodology, validation, formal analysis, investigation, writing – review and editing, visualization. **Giulia Bisson:** writing – review and editing; **Clara Comuzzi:** resources, writing – review and editing. **Daniele Goi:** conceptualization, resources, funding acquisition, writing – review and editing, supervision. **Marilena Marino:** conceptualization, validation, resources, funding acquisition, writing – review and editing, supervision, project administration.

## Conflicts of Interest

The authors declare no conflicts of interest.

## Supporting information


**Table S1:** WW physicochemical characteristics before and after sterilization.
**Figure S1:** Ozone reactor design. A—ozone generator, B—reactor containing the wastewater, C—reactor containing the KI solution to capture the residual ozone.

## Data Availability

Data will be made available on request.
