# Supplementary material for: Evaluating Low‐Frequency Ultrasound as a Pretreatment to Improve Ozonation Antimicrobial Efficacy in Urban Wastewater Treatment
Source: Water Environ Res. 2026 Feb 25;98(3):e70322. doi: 10.1002/wer.70322 (PMC12935695; doi:10.1002/wer.70322)
Supplement: Supplementary file 1 — Table S1: WW physicochemical characteristics before and after sterilization. Figure S1: Ozone reactor design. A—ozone generator, B—reactor containing the wastewater, C—reactor containing the KI solution to capture the residual ozone. [file WER-98-e70322-s001.docx]

**Supplementary material**

Table S1: WW physicochemical characteristics before and after sterilization

| Parameter | Untreated WW | Sterilized WW |
| --- | --- | --- |
| Chemical oxygen demand [mg/L] | 231.51 ± 42.77 | 187.17 ± 26.73 |
| Specific ultraviolet absorbance [L/mg m] | 1.2 ± 0.02 | 1.56 ± 0.03 |
| Total nitrogen [mg/L] | 36 ± 2.01 | 26.68 ± 1.93 |
| Total phosphates [mg/L] | 4.6 ± 0.21 | 2.3 ± 0.17 |
| Anionic surfactants [mg/L] | 7.25 ± 2.32 | 6.83 ± 1.18 |
| pH | 7.01 ± 0.3 | 6.8 ± 0.2 |

**
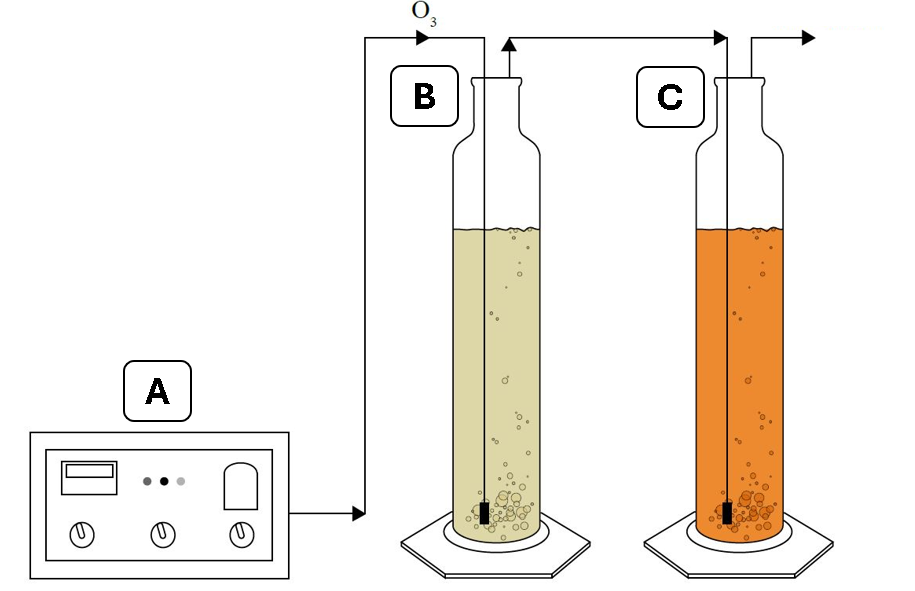
**

Figure S1: Ozone reactor design. A – ozone generator, B – reactor containing the wastewater C- reactor containing the KI solution to capture the residual ozone.
